# Supplementary material for: Inhibition of neuronal FLT3 receptor tyrosine kinase alleviates peripheral neuropathic pain in mice
Source: Nat Commun. 2018 Mar 12;9:1042. doi: 10.1038/s41467-018-03496-2 (PMC5847526; doi:10.1038/s41467-018-03496-2)
Supplement: Supplementary file 1 — Supplementary information [file 41467_2018_3496_MOESM1_ESM.pdf]

Supplementary Information

Inhibition of neuronal FLT3 receptor tyrosine kinase alleviates peripheral neuropathic pain in mice

Cyril Rivat[1,2,7], Chamroeun Sar[1,2,7], Ilana Mechaly[1,2,7], Jean-Philippe Leyris[1,4,7], Lucie Diouloufet[1], Corinne Sonrier[1,4], Yann Philipson[3], Olivier Lucas[1], Sylvie Mallié[1,2], Antoine Jouvenel[1,2], Adrien Tassou[1,2], Henri Haton[1,2], Stéphanie Venteo[1], Jean-Philippe Pin[5], Eric Trinquet[6], Fabienne Charrier-Savournin[6], Alexandre Mezghrani[1], Willy Joly[1], Julie Mion[1], Martine Schmitt[3], Alexandre Pattyn[1], Frédéric Marmigère[1], Pierre Sokoloff[4], Patrick Carroll[1], Didier Rognan[3] & Jean Valmier[1,2]

[1]Institute for Neurosciences of Montpellier, INSERM, Institut National de la Santé et de la Recherche Médicale, UMR1051, Hôpital Saint-Eloi, Montpellier, France. [2]Université de Montpellier, Montpellier, France. [3]Laboratoire d'Innovation Thérapeutique, UMR7200 CNRS-Université de Strasbourg, Illkirch, France. [4]Biodol Therapeutics, Cap Alpha, Clapiers, France. [5]Institut de Génomique Fonctionnelle, CNRS, INSERM, Univ. Montpellier, 34094 Montpellier, France. [6]Cisbio Bioassays, Parc Marcel Boiteux, BP84175, 30200 Codolet, France. [7]These authors contributed equally to this work. Correspondence should be addressed to D.R. (rognan@unistra.fr) or J.V. (jean.valmier@umontpellier.fr).

Supplementary Figures: 9
Supplementary Tables: 4
Supplementary Notes: 1
Supplementary Methods: 1
Supplementary References: 6

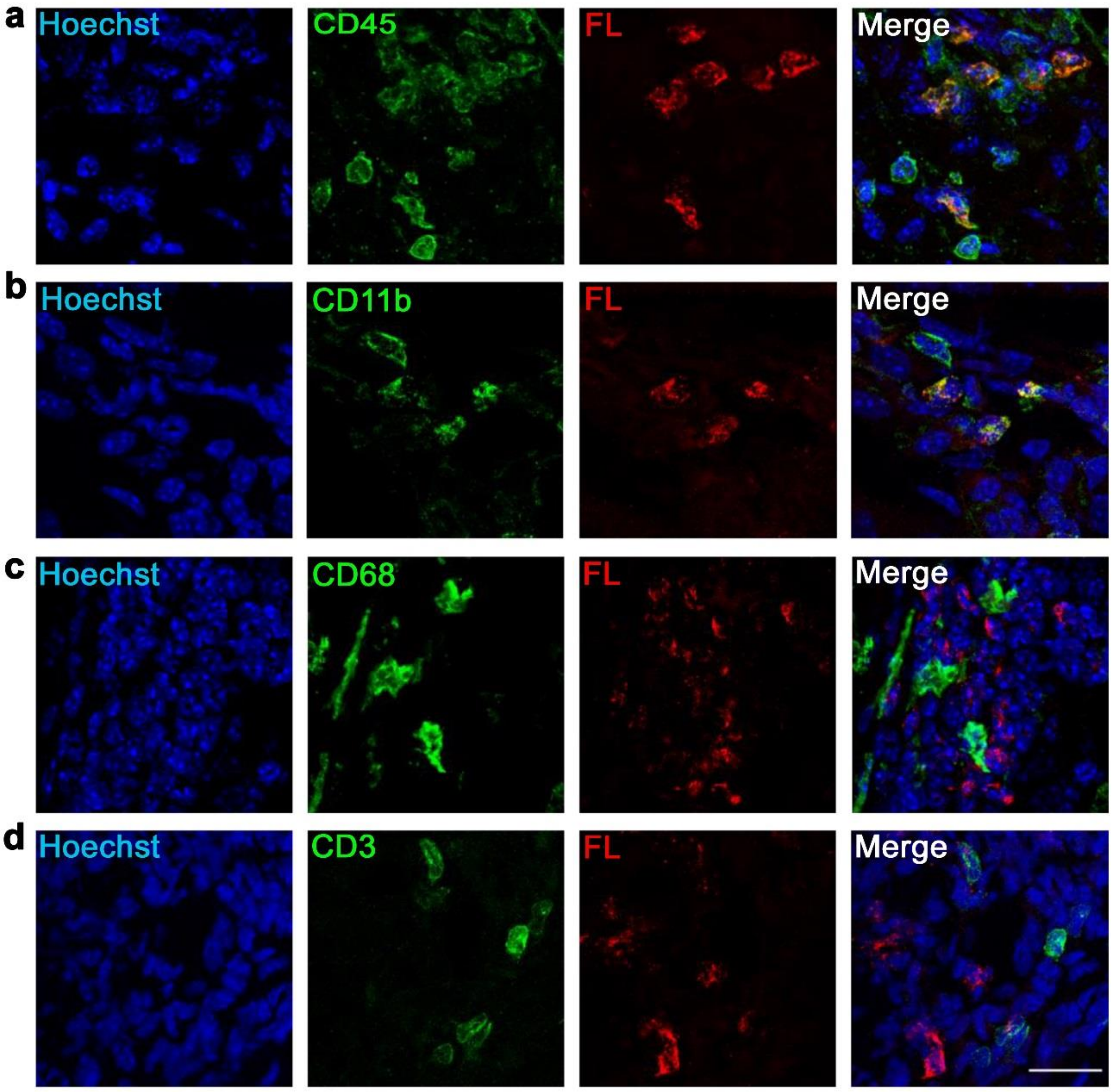

Supplementary Figure 1. Phenotypical characterization of FL-expressing cells present in the sciatic nerve at the lesion site 10 days after CCI. Cells were stained with the Hoechst dye to label the nuclei, or with antibodies against FL or markers of immune cells: **(a)** CD45, leucocytes; **(b)** CD11b, monocytes/macrophages; **(c)** CD68, macrophages; **(d)** CD3, lymphocytes T. Scale bar = 20 μM.

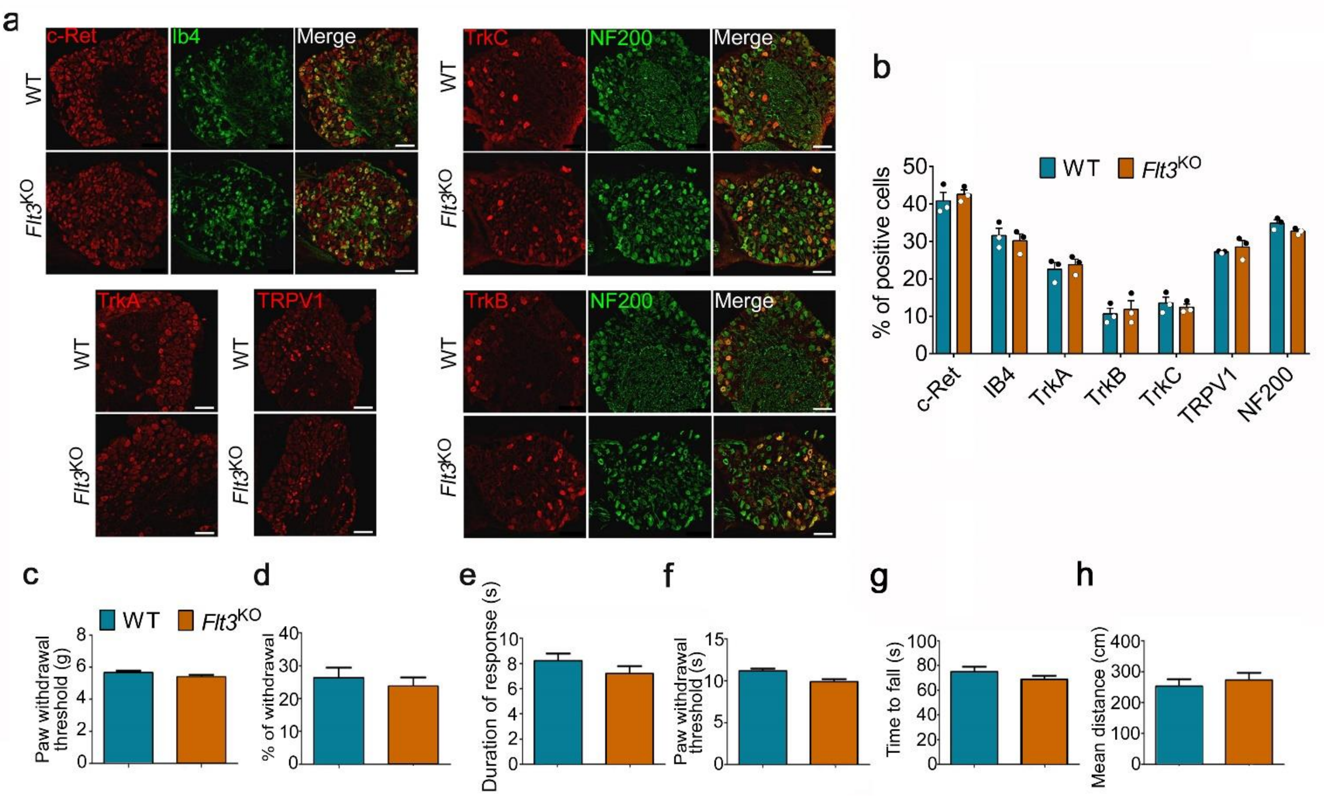

Supplementary Figure 2. *Flt3*-knock-out (*Flt3*^{KO}) mice have normal proportions of sensory neurons and express normal nociceptive and motor behaviors. (**a**) The different subpopulations of the primary sensory neurons of the DRG were immunostained with c-Ret, IB4, TrkA, TrkB, TrkC, TRPV1 or NF-200 antibodies. (**b**) Cells positive with the respective markers, were quantified as percentage of total cell number. All the values are means ± s.e.m. (*n* ≥ 6 determinations on 3 animals of each genotype). No statistical differences between genotypes were found (Mann-Whitney tests). Scale bars = 100 µm. (**c-h**) *Flt*3^{KO} and wild-type (WT) littermates show no statistical difference (unpaired Student's t-test) in tactile sensitivity evaluated in the dynamic Von Frey test (**c**), punctate mechanical sensitivity evaluated by application of 0.6 g von Frey filaments (**d**), cold thermal sensitivity evaluated by the duration of the response after acetone application on one hindpaw (**e**), heat nociceptive threshold evaluated by the Hargreaves test (**f**), balance performance in the rotarod test (**g**), and locomotor activity evaluated by the total distance performed in the open-field (**h**). In **c-h**, values are means ± s.e.m. (*n* = 8).

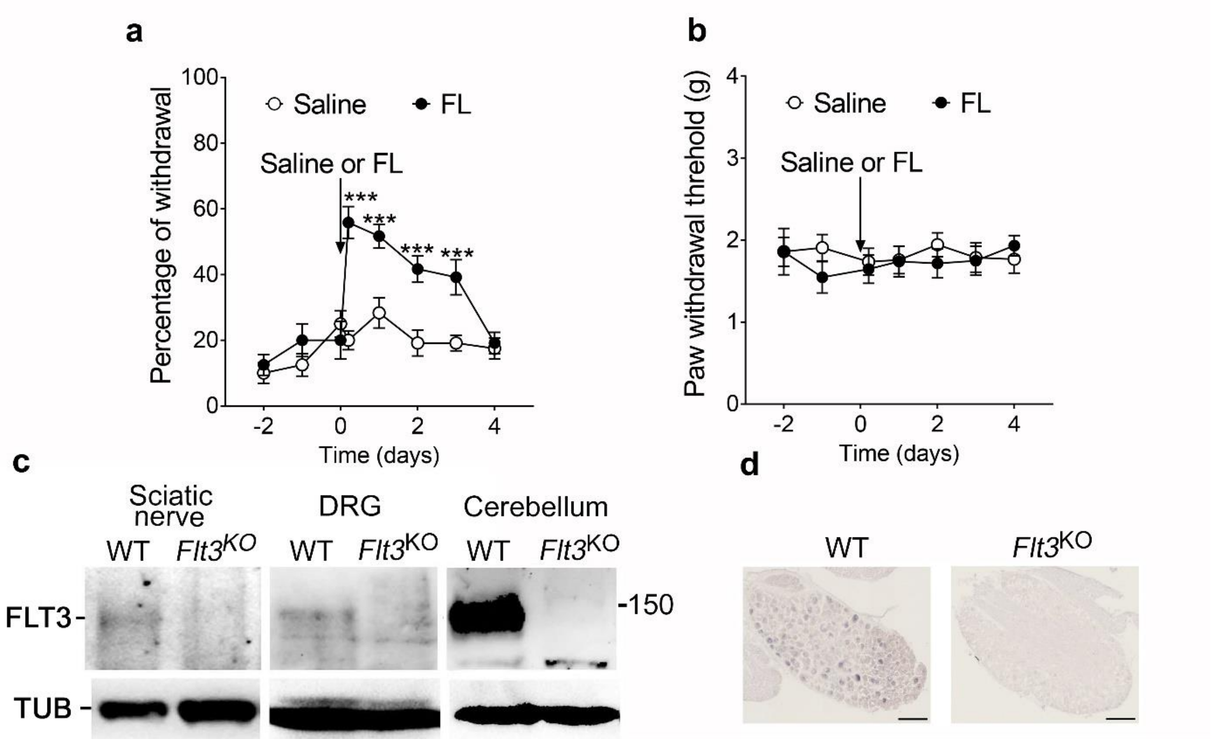

Supplementary Figure 3. Localization of the site of action of FL. (**a**) Administration of FL (50 ng/1 µl) into the sciatic nerve produced an increase in the percentage of paw withdrawal in response to 0.6 g von Frey filament (n=12) **P* < 0.05 vs. Saline by two-way ANOVA for repeated measures followed by Dunnett's test. (**b**) Intravenous injection of FL (5 µg/100 µl) did not change the mechanical nociceptive threshold. No statistical difference between animals injected with FL and saline. All the values are means ± s.e.m. (*n* = 8). (**c**) Western blot analysis showing that FLT3 is detected, as bands of expected size around 150 kDa, in the sciatic nerve, DRG and cerebellum of wild-type (WT), but not *Flt3* knock-out (*Flt3*^{KO}) mice. TUB, tubulin. (**d**) Expression of *Flt3* mRNA was detected by *in situ* hybridization in 20-25% of primary sensory neurons, characterized by their typical morphology, in the DRG of WT, but not *Flt3*^{KO} mice. Scale bars = 150 µm.

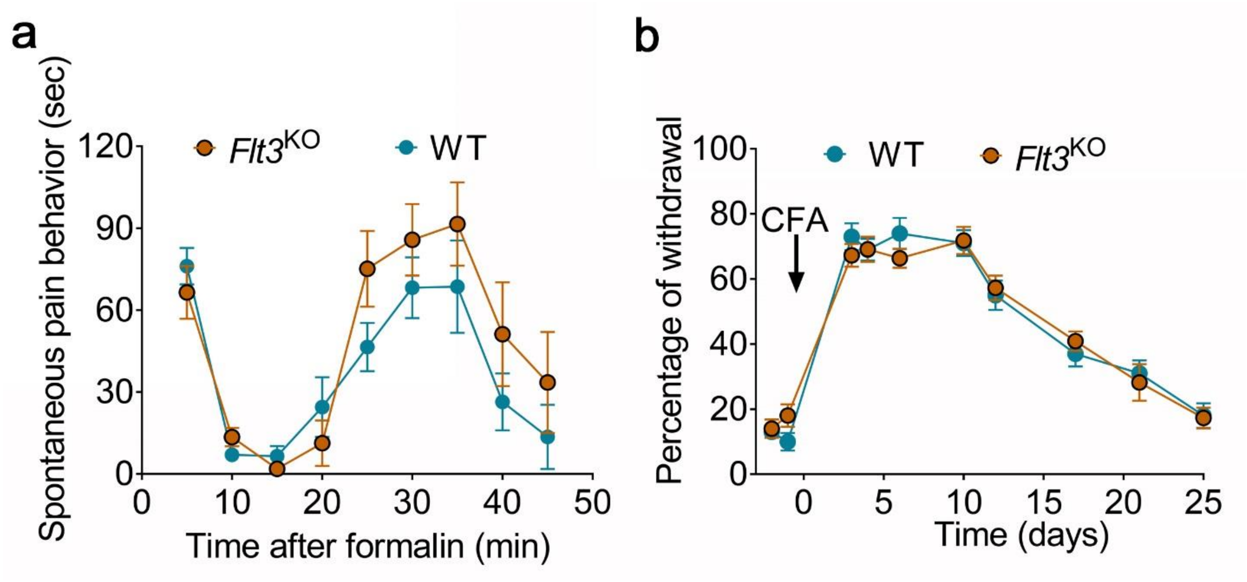

Supplementary Figure 4. Mice have normal response to acute and chronic inflammatory pains (**a, b**) *Flt*3^{KO} and wild-type (WT) littermates show no statistical difference (two-way ANOVA) in acute nociceptive and inflammatory pain in the formalin model (**a**) and in chronic inflammatory pain after paw injection with CFA (**b**). In **a-b**, values are means ± s.e.m. (n = 8).

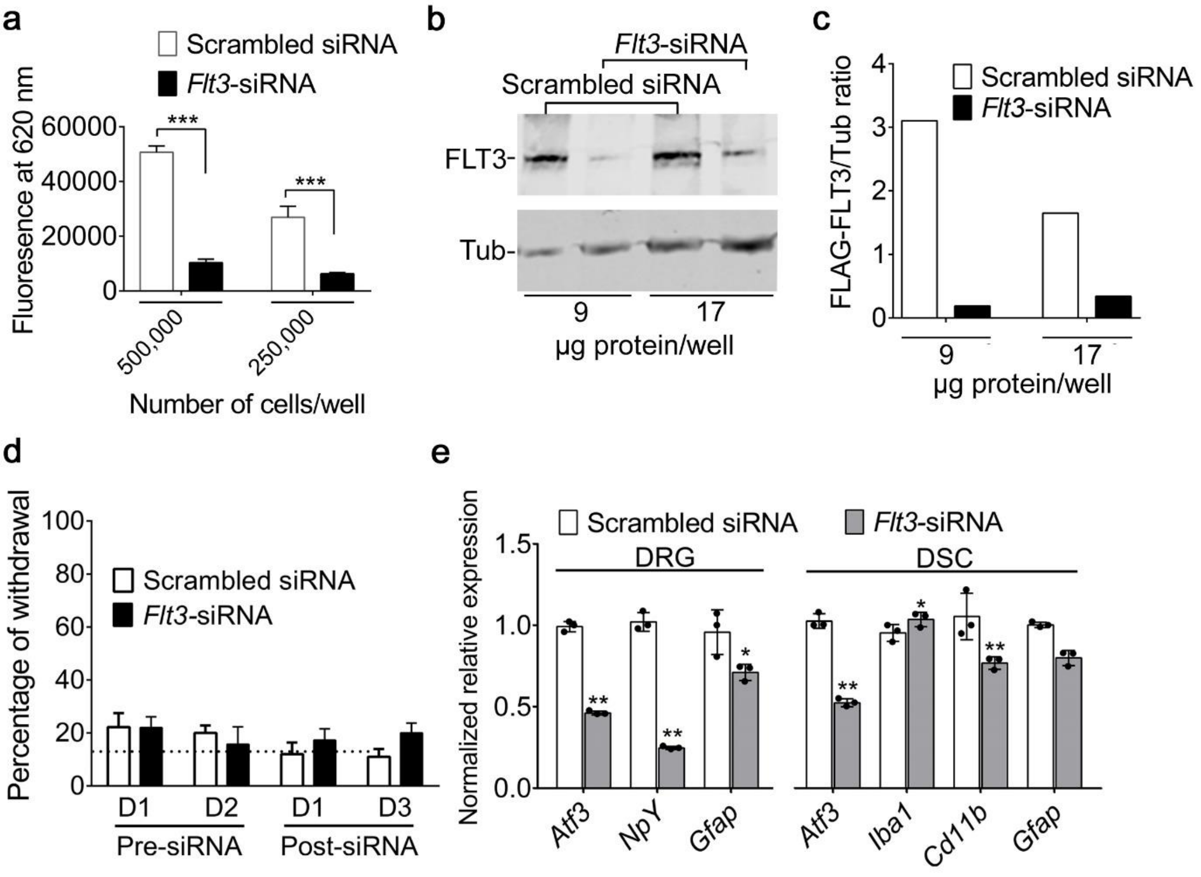

Supplementary Figure 5. An siRNA directed against *Flt3* (*Flt3*-siRNA) effectively reduces FL3 expression *in vitro* and function *ex vivo***.** (**a-c**) *Flt3*-siRNA downregulated FLT3 expression, compared to scrambled siRNA, when transfected to HEK293M cells together with a SNAP-tagged *Flt3.* FLT3 was quantified by SNAP fluorescence with Tb-labelled benzylguanine (**a**) and by Western blot (**b**), which was quantified in (**c**). In **a**, ***P < 0.001 by unpaired Student's t-test (n = 3). (**d**) *Flt3*-siRNA did not change pain sensitivity in non-injured animals. Two pain sensitivity baselines were measured at days 1 and 2 (D1 and D2, Pre-siRNA). Then *Flt3*-siRNA or scrambled siRNA was infused intrathecally and mechanical nociception was measured 1 and 3 days after injection (D1 and D3, Post-siRNA). Values are means ± s.e.m. of data from 8 animals. No statistical difference between *Flt3* siRNA- and scrambled siRNA-treated animals by ANOVA with repeated measures. (**e**) Intrathecal infusion of *Flt3*-siRNA reduces PNP-related gene expressions in the DRG and DSC. Normalized relative expression of PNP-related biomarkers mRNA, measured by q-PCR after a 14-days infusion of *Flt3*-siRNA starting six days after CCI. The *Flt3*-siRNA reduced expressions of *Atf3*, *NpY*, and *Gfap* in the DRG and of *Atf3*, the microglial markers *Iba1* and *Cd11b* in the DSC, as compared with scrambled siRNA infusion. All the values are means ± s.e.m. of data from 3 animals. *P < 0.05; **P < 0.01 vs. Scrambled siRNA by unpaired Student's t-test.

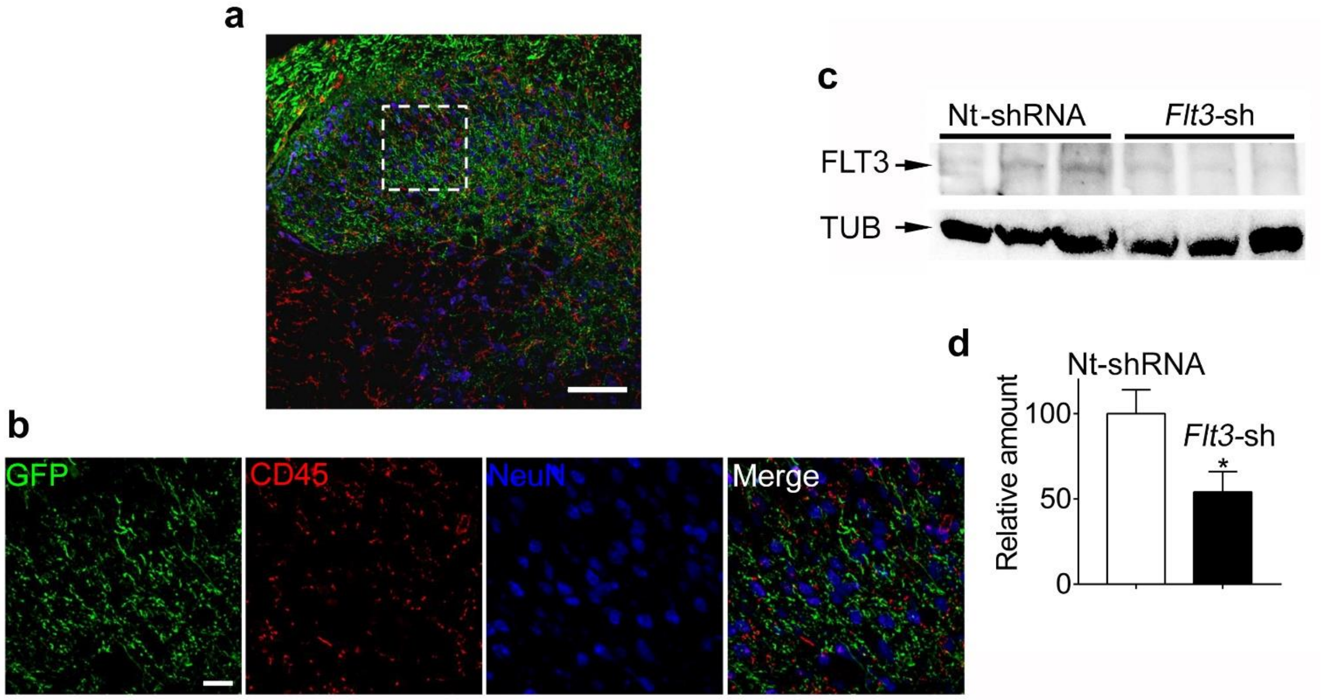

Supplementary Figure 6. *Flt3*-targeted shRNA reduces the expression of FLT3 in the DRG. (**a**) Expression of the AAV9 virus expressing anti-*Flt3* shRNA and GFP in the DSC. Bar = 100 µm. (**b**) The enlargement of the dotted area in (**a**) shows that the virus-encoded GFP was not localized in CD45-positive cells or neurons, as labelled by NeuN, but on fibers. Bars = 20 µm. (**c**) Western blot of DRG tissue extracted from animals receiving an intrathecal injection of an AAV9-GFP expressing an *Flt3*-targeted shRNA (*Flt3*-sh) or a non-targeting shRNA (Nt-shRNA). Tubulin (TUB) serves as standard. (**d**) Quantification of (**c**). Values are means ± s.e.m. (n = 7-8 determinations). *$P < 0.05$ vs. Ir-shRNA by unpaired Student t-test.

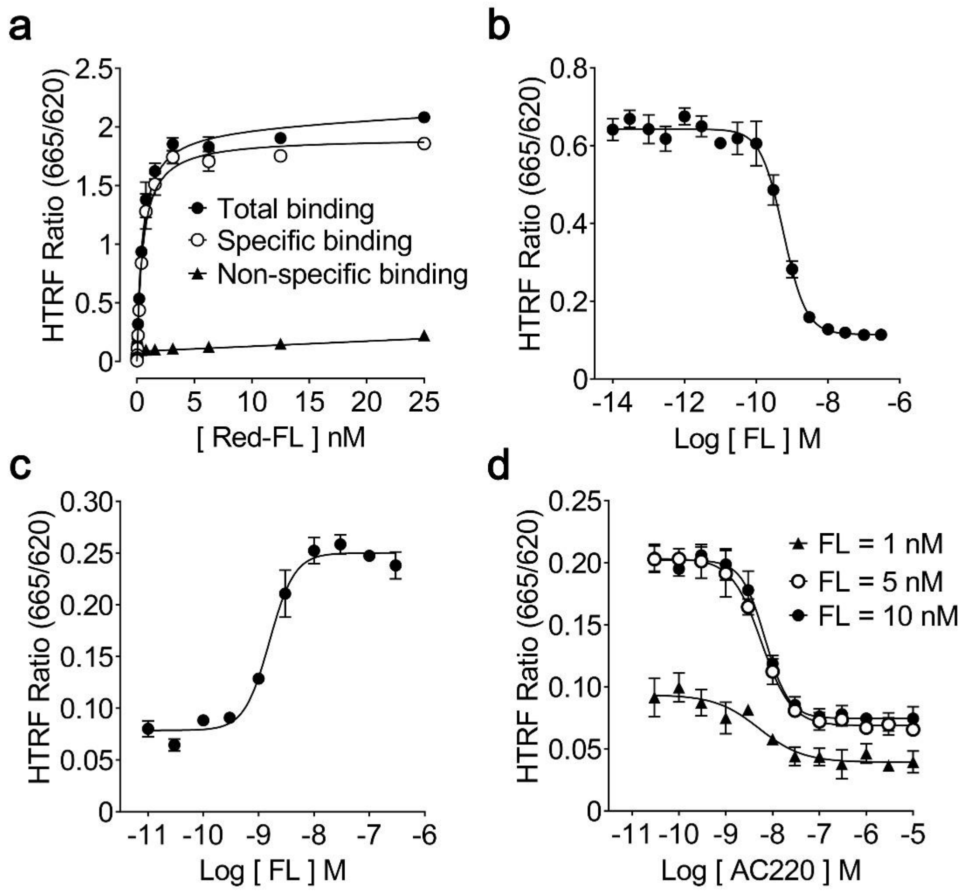

Supplementary Figure 7. FLT3 binding and autophosphorylation measured by time-resolved Fluorescence Energy Transfer (trFRET). (**a**) Saturation of Lumi4-Tb-SNAP-FLT3 binding by Red-FL in HEK cells. Homogeneous Time Resolved Fluorescence (HTRF) signal was measured between a fluorescent donor probe (Lumi4-Tb cryptate) located on a SNAP-tagged2 FLT3 receptor and a fluorescent acceptor probe (Red) located on a recombinant dimeric FL ligand. Non-specific binding was measured in the presence of 1 µM FL. (**b)** Displacement of Red-FL (0.5 nM) by unlabeled FL, with an IC_{50} of 0.57 nM. (**c**) Stimulation by FL of autophosphorylation of FLT3 at Y969, with an EC_{50} of 1.6 nM. Homogeneous Time Resolved Fluorescence (HTRF) signal was generated between a first antibody (8F2 Mab) labelled with a Lumi4-Tb donor probe (recognizing an intracellular FLT3 epitope nearby Ser740), and a second antibody (PY969) labeled with a d2 acceptor probe (specifically recognizing phosphorylated FLT3 at Tyr969). (**d)** Inhibition of FL-induced FLT3-Y969 phosphorylation by an intracellular FLT3 inhibitor (AC220, also known as quizartinib), with IC_{50} of 5.2, 5.8 and 7.0 nM at FL concentrations of 1, 5 and 10 nM, respectively. Results are expressed as the ratio between fluorescence at 665 and 620 nm and are means ± s.e.m. of quadruplicate determinations. The experiments were replicated twice, with identical results.

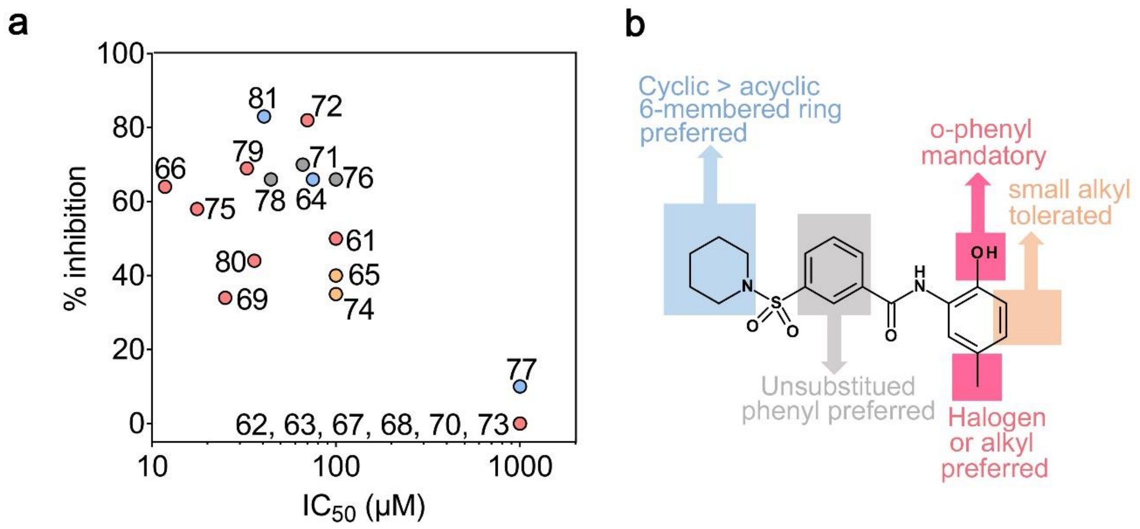

Supplementary Figure 8. FLT3 inhibition by commercial analogs of compound **3**. (**a**) Half maximum inhibitory concentration (IC_{50}; inhibition of Red-FL binding to Lumi4-Tb-SNAP-FLT3 overexpressed in HEK293 cells) as a function of the maximal percentage of inhibition at a competitor concentration of 10^{-3}M. Colors refer to chemical modifications in (**b**). (**b**) Preliminary structure-activity relationships. The ortho-phenol moiety seems to be mandatory for FLT3 binding since its deletion (compounds **68**, **74**, **77**), displacement to the meta position (compounds **62**), or methylation (compound **69**) increase IC_{50} values. Whereas apolar substituents (alkyl, chloride) are clearly preferred in position 4 of the terminal aromatic ring (compounds **66** and **75**), an unsubstituted central phenyl ring seems to be preferred. Last, a cyclic secondary amine (piperidine, azepane) was clearly preferred to an acyclic amine (compound **64**) at the conserved sulfonamide moiety.

a

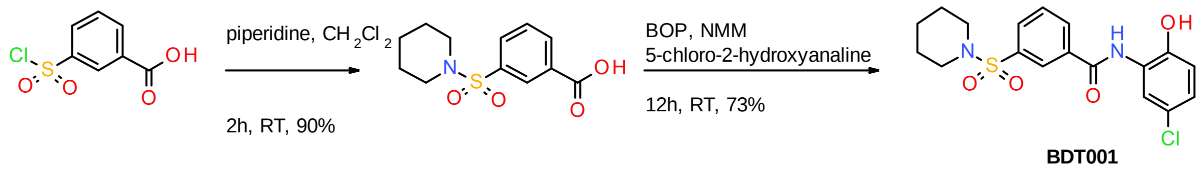

b

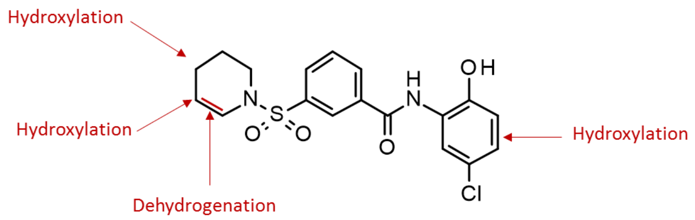

Supplementary Figure 9. Synthetic access to (**a**) and main metabolites of BDT001 (**b**), identified by LC-MS/MS after 1h incubation at 37°C with mouse liver microsomes (5 mg/ml), 1 mM NAPDH, 3mM $MgCl_2$ in phosphate buffer ($t_{1/2}$ = 7 min, $CL_{h,nt}$ = 188 ml/min/kg).

Cpd	Fit[a]	Pharmprint[b]	Structure	Cpd	Fit[a]	Pharmprint[b]	Structure
1	3.17	11111	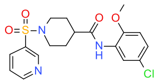	15	3.01	01111	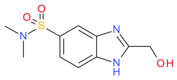
2	3.20	01111	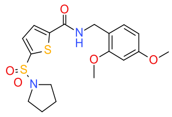	16	3.27	01111	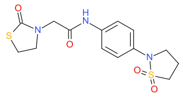
3	3.32	01111	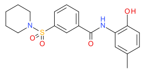	17	3.48	01111	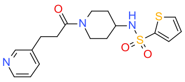
4	3.32	01111	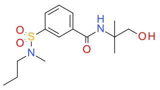	18	3.04	01111	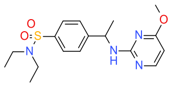
5	3.50	01111	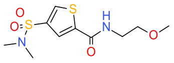	19	3.17	11101	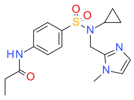
6	3.27	11101	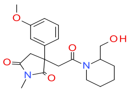	20	3.02	01111	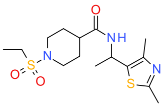
7	4.08	11111	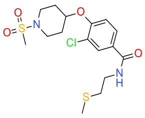	21	3.23	01111	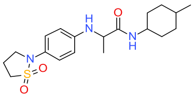
8	3.04	01111	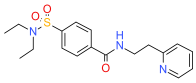	22	3.20	01111	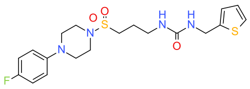
9	3.43	01111	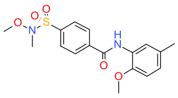	23	3.06	01111	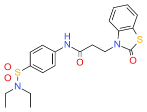
10	3.43	01111	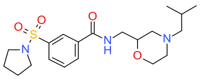	24	3.30	01111	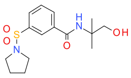
11	3.26	01111	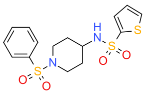	25	3.04	01111	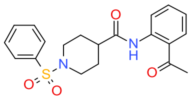
12	3.16	01111	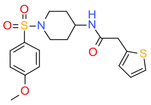	26	3.21	01111	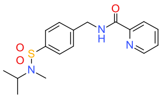
13	3.12	01111	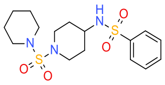	27	3.07	01111	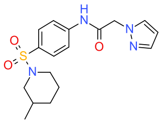
14	3.24	01111	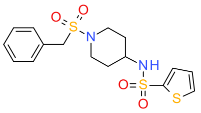	28	3.22	01111	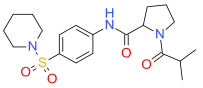

Supplementary Table 1 Structure of virtual hits. [a] Pharmacophore fitness value defined as: $Fit = \sum_f (1 - SSE); SSE = \left(\frac{D}{T}\right)^2$ where D is the distance in Å of the mapped atom to the feature (f) center, and T is the radius in Å of the feature sphere.[b] For each pharmacophoric feature, a bit is switched 'on" or 'off' as whether the feature is present in the aligned ligand. Features are sorted as follows: h-bond acceptor1 (FL:His8 O atom), h-bond acceptor 2 (FL:Ser9 O atom), h-bond acceptor3 (FL: Pro10 O atom), h-bond acceptor 4 (FL: Ser13 OG atom), h-bond donor 5 (FL: Ser13 N atom).

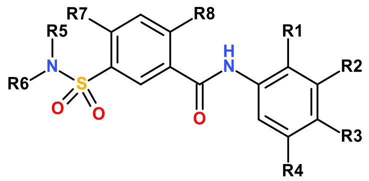

Compound	R1	R2	R3	R4	R5	R6	R7	R8
3	OH	H	H	CH_3	piperidine		H	H
61	CH_3	H	H	H	piperidine		H	H
62	H	OH	H	H	piperidine		H	H
63	H	H	CH_3	H	piperidine		H	H
64	OH	H	H	H	CH_2CH_3	CH_2CH_3	H	H
65	OH	H	H	CH3	pyrrolidine		H	H
66	OH	H	H	Cl	piperidine		H	H
67	OH	H	H	H	piperidine		OCH_3	H
68	H	H	$CONH_2$	H	pyrrolidine		H	H
69	OCH_3	H	H	H	piperidine		H	H
71	OH	H	H	H	piperidine		H	Cl
72	OH	H	H	H	piperidine		H	H
74	H	H	SO_2CH_3	H	piperidine		H	H
75	OH	H	H	*t*-Bu	piperidine		H	H
76	OH	H	H	H	piperidine		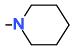	H
77	H	H	$COCH_3$	H	pyrrolidine		H	H
78	OH	H	CH_3	H	piperidine		H	H
79	OH	H	H	CH_3	piperidine		CH_3	H
80	OH	H	H	H	piperidine		CH_3	H
81	OH	H	H	H	azepane		H	H

70

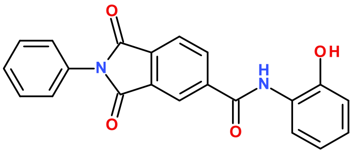

73

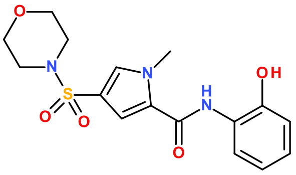

Supplementary Table 2 Commercial analogues of hit 3.

Receptor Tyrosine Kinase	Reference Ligand	% Inhibition of reference binding
c-KIT	SCF	7.8
c-Ret-GFRα2	Neurturin	-4.1
DDR1	Collagen II	-0.7
EphA5	Ephrin A3	5
EphA7	Ephrin A3	4.3
EphB2	Ephrin B2 (murine)	1.4
EphB3	Ephrin B2 (murine)	2.8
EphB4	Ephrin B2 (murine)	22.7
ErbB1	EGF	8.7
ErbB2-ErbB3	Heregulin-β1	13.6
ErbB4	NGR1	-0.1
FLT4	VEGF	16.8
IGF1R	IGF-1	10.1
INSRb	Insulin (Human)	10
JAK3	Prolactin	16.7
KDR	VEGF-121	-3.1
PDGFRa	PDGF-BB	2.2
PDGFRb	PDGF-BB	4.2
PRLR-JAK1	Prolactin	6.1
PRLR-JAK2	Prolactin	1.9
TrkA-P75	β-NGF	13.7
TrkB-P75	BDNF	-1.3
TrkC-P75	NT-3	0.6

Supplementary Table 3 Binding of BDT001 (10μM) to 23 receptor tyrosine kinases (PathHunter eXpress functional Assay, DiscoverX, ref *tk*MAX).

Kinase	Reference Compound	% Inhibition of reference binding	Kinase	Reference Compound	% Inhibition of reference binding
Abl kinase	staurosporine	16	JNK1	staurosporine	32
Akt1/PKBα	staurosporine	-13	KDR kinase	staurosporine	-6
AurA/Aur2 kinase	staurosporine	-5	Lck kinase	staurosporine	-14
CaMK2α	AIP	6	MAPKAPK2	staurosporine	6
CDC2/CDK1	staurosporine	26	MARK1	staurosporine	13
CDK2	staurosporine	33	MNK2	staurosporine	-7
CHK1	staurosporine	-10	NEK2	staurosporine	-23
CHK2	staurosporine	6	p38α kinase	SB202190	23
c-kit kinase	staurosporine	-1	PAK2	staurosporine	-8
c-Met kinase	staurosporine	0	PAK4	staurosporine	6
EGFR kinase	PD153035	4	PDGFRα kinase	staurosporine	40
EphA2 kinase	staurosporine	-[a]	PDGFRβ kinase	staurosporine	-2
EphA3 kinase	staurosporine	0	PDK1	staurosporine	3
EphB4 kinase	staurosporine	-[a]	Pim2 kinase	staurosporine	19
ERK	staurosporine	11	PKA	staurosporine	7
FGFR1 kinase	staurosporine	-7	PKCbeta 2	staurosporine	1
FGFR2 kinase	staurosporine	-2	PLK1	staurosporine	-5
FGFR3 kinase	staurosporine	33	RAF-1 kinase	staurosporine	11
Fms/CSFR kinase	staurosporine	1	ROCK1	staurosporine	2
GSK3β	staurosporine	0	SGK1	staurosporine	16
HGK	staurosporine	-[a]	SIK	Ro-318220	-6
IKKα	staurosporine	20	Src kinase	staurosporine	-4
IRAK4	staurosporine	64	TAOK2	staurosporine	33
IRK	staurosporine	-4	TRKA	staurosporine	0
JAK3	staurosporine	-3			

Supplementary Table 4 Intracellular inhibition of 49 kinases (ExpresS Diversity kinase profile, Eurofins CEREP, ref P10) by BDT001 at a concentration of 10 µM.
[a] Test compound interferes with the assay detection method.

Supplementary Note 1:

The discrepancy observed between FLT3-inhibiting concentrations of BDT001 in different cellular environments (10-20 µM in RS4-11 or HEK293 cells expressing SNAP-tagged FLT3, submicromolar in DRG neuronal FLT3) is a hallmark of the other two extracellular receptor tyrosine kinase inhibitors (ANA-12, SSR128129E) known to date, targeting Tropomyosin Receptor Kinase B (TrkB) and Fibroblast Growth Factor (FGF) receptor, respectively[1, 2]. Discovered by primary screens designed for identifying orthosteric competitive inhibitors, the two compounds allosterically prevent the recognition of an endogenous ligand (BDNF and FGF2) to TrkB and FGFR receptors, respectively. IC_{50} values of these two compounds in various *in vitro* assays is highly dependent on the cellular context. For example, ANA-12 exhibits both a low affinity (IC_{50} = 40 µM) and high affinity site (IC_{50} = 40 nM), the latter being mainly observed in neuronal cells and attributed to the presence of a co-receptor ($p75^{NTR}$) stabilizing an ANA-12-preferred conformational state of the TrkB receptor. Likewise, SSR128129E inhibits ^{125}I-FGF2 binding to the extracellular domain of FGFR1 with a half-maximal inhibitory concentration of 1.9 µM, whereas it inhibits FGF2-induced HUVEC endothelial cells proliferation and migration at much lower concentrations (IC_{50} = 15 and 30 nM,[2] respectively).

Although we have not characterized yet the high affinity-binding site of BDT001 at the FLT3 receptor, several explanations to the above-noted discrepancy are possible: (i) The conformational arrangement of the FLT3 extracellular D1-D5 domains may be unique to neuronal cells. Intriguingly, the crystal structure of the FLT3-FL complex[3], which guided the discovery of BDT001, does not exhibit the prototypical inter-chain contacts (notably at the D4 domain) observed in other cytokine-RTK structures (e.g. KIT-SCF, CSF1R-CSF)[4]. In the FLT3-FL structure, the two membrane proximal D5 domains are too far away (20 Å) to promote the dimerization of the missing transmembrane domains. It is possible that the particular experimental settings used to generate the FLT3-FL crystals have led to a partially activated FLT3 structure different from that targeted by BDT001 in DRG neurons, (ii) the high-affinity binding site of BDT001 may be only unmasked in neuronal cells and depend on the presence of a yet unknown co-receptor stabilizing a peculiar apostructure of the FLT3 receptor. Elucidating structural and functional features of the interaction between BDT001 and neuronal native FLT3 requires the setup of more physiological assays, complying with the context-dependence of this interaction. Indeed, DRG neurons seems an adequate model, for which, however, highly sensitive techniques are needed given the limited number of these neurons.

In spite of the difficulty, allosteric RTK modulators are attractive from a therapeutic perspective[5], because they are expected to be more selective and safer than classical intracellular inhibitors targeting the highly conserved ATP-binding domain.

Supplementary Methods

Chemicals and reagents

Virtual hits and analogues were purchased from six commercial suppliers (Asinex, Chembridge, Chemdiv, Enamine, Lifechemicals, Uorsy) and their purity checked by LC-MS using an Agilent 1200SL series system. Samples were injected onto an Agilent 1200SL 1.0 mm × 30 mm, 1.9 µM Hypersil Gold Thermo column at room temperature. A mobile phase of A being H_2O + 0.05% formic acid and B being acetonitrile was used with a linear gradient from 2% to 95% B in 6.0 min. Mass spectral data were acquired in MM-ESI/APCI + and - mode. The purity of all compounds was above 90%.

The Tag-lite labeling medium was from Cisbio Bioassays (Ref. LABMED). The 96 well plates (Ref.655086) as well as the 384 wells small volume plates (Ref.784075 and 784076) were from Greiner Bio-One. The Lumi4-Tb derivative of O6-Benzylguanine was synthesized by Cisbio Bioassays and is commercialized as SNAP-Lumi4-Tb (Cisbio Bioassays, Ref. SSNPTBE).

A recombinant rh-FLT3-L was produced as previously reported[6] and labeled with a red fluorescent probe (Red-FL) by Cisbio in collaboration with the Institut des Neurosciences de Montpellier. Poly-L-ornithine (MW of 30,000-70,000 daltons) was from Sigma-Aldrich. The plasmid encoding for the SNAP tagged human FLT3 (pSNAP-FLT3) used for transient transfection was obtained from Cisbio Bioassays. Monoclonal antibodies against FLT3 (8F2) and phosphotyrosine FLT3 (PY969) were purchased from Cell Signaling Technology and labeled with Lumi4-Tb and d2 respectively, by Cisbio.

Synthesis of BDT001

Piperidine (0.35 g, 0.4ml, 4.08 mmol) was added to a stirred solution of 3-(chlorosulfonyl) benzoic acid (0.3 g, 1.36 mmol) in CH_2Cl_2 (5 ml) at 0° C, and the resulting solution was stirred for 2h. The volatiles were then evaporated under reduced pressure and the residue treated with aqueous 1N $KHSO_4$. The aqueous phase was then extracted with ethyl acetate (×3), and the combined organic phases dried over anhydrous magnesium sulfate, filtered and evaporated in vacuo to give 3-(piperidin-1-ylsulfonyl)benzoic acid as a white solid (0.34 g, 1.262 mmol, 90%). ^{1}H NMR (400MHZ, $CDCl_3$): δ (ppm) 8.41 (1H, br), 8.26 (1H, dt, *J* = 8.0Hz , 1.4 Hz), 7.94 (1H, dt, J= 8.0 Hz , 1.6 Hz), 7.61 (1H, t, J= 7.7 Hz), 2.98-2.96 (4H, m), 1.62-1.56 (4H, m), 1.40-1.34 (2H, m).^{13}C NMR (100 MHz, $CDCl_3$): δ (ppm) 170.0, 137,6, 133.9, 132.5, 130.3, 129.4, 129.2, 46.9, 25.2, 23.5.

(Benzotriazol-1-yloxy)tris(dimethylamino)phosphonium hexafluorophosphate (BOP, 0.29 g, 0.668 mmol), N-Methyl-morpholine (0.14 g, 1.39 mmol) was added to a stirred solution of the obtained 3-(piperidin-1-ylsulfonyl) benzoic acid (0.15 g, 0.557 mmol) in dichloromomethane (3.15 ml) at room temperature, and the resulting solution was stirred for 15min. 5-chloro-2-hydroxyaniline (0.080g, 0.557 mmol) was then added and the final mixture was stirred at room temperature for 12h. The solvent was then evaporated under reduced pressure, the residue taken into ethyl acetate (15 ml) and washed with HCl 1N (8 ml), $KHCO_3$ 1N (8 ml), and water (10 ml). The organic layer was dried (Na_2SO_4), filtered and concentrated under reduced pressure. The resulting residue was purified by silica gel flash chromatography (AcOEt/heptane: 3/7) to yield *N*-(5-chloro-2-hydroxyphenyl)-3-(piperidin-1-ylsulfonyl) benzamide (**BDT001**) as a white solid (160 mg, 73%). ^{1}H NMR (400MHz, d6-DMSO) δ (ppm) 10.08 (s, 1H), 9.90 (s, 1H), 8.28 (s, 1H), 8.27 (s, 1H), 7.93 (dt, 1H, J = 8 – 1.2 Hz), 7.81 (t, 1H, J = 8 Hz), 7.73 (d, 1H, J = 2.1 Hz), 7.11 (dd, 1H, J = 8 – 2.8 Hz), 6.95 (d, 1H, J = 8 Hz), 2.94

(t, 4H, J = 5.2), 1.56 (m, 4H), 1.38 (m, 2H). ^{13}C NMR (100 MHz, $CDCl_3$): δ (ppm) 166.5, 146.9, 137.3, 134.4, 132.1, 131.1, 129.9, 126.9, 126.5, 126.1, 125.5, 122.5, 120.0, 47.0, 25.05, 23.3. HRMS-ESI (m/z) $[M+H]^+$: calculated for $C_{18}H_{20}ClN_2O_4S$ 395.0832, found 395.0827.

Cell culture

HEK293T cells, used for binding assays, were maintained in DMEM Glutamax (Invitrogen) supplemented with antibiotics (penicillin 50 U/ml, streptomycin 50 µg/ml) and 10% heat-inactivated Foetal Calf Serum. The HEK 293 T cells do not express the FLT3 receptor, and were transiently transfected with human *Flt3* receptor cDNA (see below). RS4-11 cells, used for auto-phosphorylation assays since they constitutively express FLT3 receptor, were maintained in RPMI-1640 (Invitrogen) and 10% Foetal Calf Serum.

Transfection procedures

Transfections were performed in 96-well plates using a cell density of 50,000 cells per well. Prior to cell plating, wells were pre-coated with Poly-L-Ornithine (50 µl of 10 mg/ml) for 30 min at 37°C. Transfection mixes were prepared using 100 ng of SNAP-FLT3 plasmid, 0.25 µl of Lipofectamine 2000 (Invitrogen) and 50 µl Opti-MEM per well. Prior to their addition to plates, transfection mixes were preincubated for 20 min at room temperature. Then 100 µl of HEK293T cells at a density of 500,000 cells/ml were plated in each well and were incubated at 37°C under 5% CO_2 for 24h.

Transient transfections were performed in batches to further generate frozen cells. HEK293T cells in complete cell medium were grown in a T75 cm^2 flask placed at 37°C under 5% CO_2. At 80% confluency, the cell medium was removed and replaced by 6 ml of fresh cell culture medium. Then, a transfection mix containing 10 µg plasmid, 25 µl lipofectamine 2000 and 4 ml Opti-MEM final volume was incubated for 20 min at room temperature prior to addition on cells. After the addition of mix, the culture flask was incubated at 37°C under 5% CO_2 for 24 h.

Covalent labeling of cells expressing the SNAP tagged-FLT3

Cell culture medium was removed from the 96-well plates and 100 nM of SNAP-Lumi4 Tb, previously diluted in the Tag-lite labelling medium, was added at 100 µl per well, and further incubated 1 h at 37°C under 5% CO_2. The excess of SNAP-Lumi4-Tb was removed by washing each well 4 times with 100 µl of Tag-lite labelling medium.

After removal of the cell culture medium from a flask containing adherent cells, 5 ml of Tag-lite labelling medium containing 100 nM of SNAP-Lumi4-Tb was added to the flask and incubated for 1 h at 37°C under 5% CO_2. The excess of SNAP-Lumi4-Tb was removed by washing each flask 4 times with 5 ml of Tag-lite labelling medium. Cells were detached, pelleted by centrifugation (5 min at 1,300 rpm) and suspended in a cell culture medium containing 10% DMSO. Then, cells were distributed at 5 million cells per vial and slowly frozen to - 80°C in isopropanol then transferred in liquid nitrogen for storage. Prior to their use, the frozen cells were thawed quickly at 37°C, the medium was removed from the vials and the cells were suspended in the Tag-lite labelling medium at a cell-density of 1 million cells/ml.

Tag-lite binding assays

Tag-lite binding assays were performed 24 h after transfection on fresh cells. When using frozen cells, binding assays were carried out immediately after cell thawing. When the assay was carried out on adherent cells in 96-well plates, the cell density was 50,000 cells per well, while a density of 10,000 cells per well was used to carry out binding assays in suspension in 384-well small volume plates.

Saturation binding experiments were performed by incubating the cells with increasing concentrations of Red-FL diluted in the Tag-lite labelling medium. For each concentration, non-specific binding was determined by adding 0.3 µM of unlabeled FL diluted in the same buffer. In plates containing labelled cells, 50 µl (or 10 µl in 384 wells plates) of Tag-lite labelling medium, 25 µl (or 5 µl for 384 well plates) of unlabeled FL or Tag-lite labelling medium were added, followed by the addition of 25 µl (or 5 µl in 384-well plates) Red-FL. Plates were incubated for 4h or overnight at room temperature before signal detection.
For competition binding assays, both Red-FL and the compounds to be tested were diluted in Tag-lite labelling medium. Cells were incubated with 0.5 nM Red-FL in the presence of increasing concentrations of compounds to be tested. In the plates containing labelled cells, 50 µl (or 10 µl in 384-well plates) of Tag-lite labelling medium, 25 µl (or 5 µl in 384-well plates) of compounds to be tested were added and incubated for 1h at Room temperature prior to the addition of 25 µl (or 5 µl in 384-well plates) of Red-FL. Plates were then incubated at room temperature for 4h or overnight before signal detection.

Auto-Phosphorylation assays

A density of 100,000 RS4-11 cells per well was used to carry out this assay in suspension in 384-well small volume plates to determine the degree of phosphorylation of FLT3 receptor.
Rh-FL was diluted in Tag-lite labelling medium. Cells were incubated in the presence of increasing concentrations of rh-FL. In the plates containing RS4-11 cells (10 µl/well), 2 µL of rh-FL were added and incubated for 10 min at room temperature. 4 µl per well of lysis buffer were added and incubated for 2 h at 4°C. A mix of anti-FLT3 and an anti-TYR-969 FLT3 antibodies labeled with Lumi4-Tb and d2 respectively (4 µL) diluted in Tag-lite labelling medium were added on cell lysate. Plates were then incubated at room temperature for 2 h or overnight before signal detection.
In competition assays, both rh-FL and the compounds to be tested were diluted in Tag-lite labelling medium. Cells were incubated with 0.1 µM of rh-FL in the presence of increasing concentrations of compounds to be tested. In the plates containing RS4-11 cells (5 µl /well), 5 µl of compounds to be tested were added and incubated for 1 h at room temperature prior to the addition of 2 µl of rh-FL at 10, 5 or 1 nM. After incubation for 3 min at room temperature with rh-FL, 4 µl of lysis buffer were added and incubated for 2 h at 4 °C. A mix of anti-FLT3 and an anti-TYR-969 FLT3 antibodies labeled with Lumi4-Tb and d2 respectively (4 µL) diluted in HEPES 50mM, were added to the cell lysate. Plates were then incubated at room temperature for 2 h or overnight before signal detection.

Signal detection

Signal was detected using an advanced fluorescence microplate reader (RUBYstar, BMG Labtech) equipped with a HTRF optic module allowing a donor excitation at 337 nm and a signal collection both at 665 nm and 620 nm. A frequency of 20 flashes/well was selected for the laser excitation. The signal was collected both at 665 nm and 620 nm using the following time-resolved settings: delay 50 µs, integration time 400 µs. HTRF ratios were obtained by dividing the acceptor signal (665 nm) by the donor signal (620 nm).

Data analysis and statistics

K_d values of the fluorescent ligand were determined from saturation curves of the specific binding using GraphPad Prism software (GraphPad Software, Inc., San Diego, CA). K_i values of the compounds were determined from binding competition experiments according to the Cheng and Prussoff equation. EC_{50} and IC_{50} values of the rh-FL and compounds were

determined from sigmoidal dose-response curves using GraphPad Prism software (GraphPad Software, Inc., San Diego, CA).

ADMET properties and in vitro safety profiling

Metabolic stability was estimated by incubating a 1µM PBS solution of the compound in 0.5 mg/ml liver microsomes (mice), 1 mM NADPH and 3 mM $MgCl_2$ at 37 °C. After 30 s homogenization, 70 µl of solution are extracted and mixed with 70 µl acetonitrile at 0°C. The same protocol was repeated after 15, 30, 45 and 60 min. Samples were stirred for 3 min in a vortex, sonicated for another 3 min and centrifuged at 15 000 g for 5 min at 4 °C. A negative control was done by replacing NADPH by an equivalent volume of the buffer and a sample taken after 60 min incubation. A positive control was carried out by incubating testosterone in the above described conditions. All samples were analyzed by LC-MS/MS as described above. ExpresS Diversity Kinase Profile (ref. P10) was performed by Eurofins CEREP (Celle L'Evescault, France). Receptor tyrosine kinase profiling (TKscan panel) was achieved by DiscoverX (Fremont, US.A.)

Supplementary References

1. Cazorla M, Premont J, Mann A, Girard N, Kellendonk C, Rognan D. Identification of a low-molecular weight TrkB antagonist with anxiolytic and antidepressant activity in mice. *J Clin Invest* **121**, 1846-1857 (2011).

2. Bono F*, et al.* Inhibition of tumor angiogenesis and growth by a small-molecule multi-FGF receptor blocker with allosteric properties. *Cancer Cell* **23**, 477-488 (2013).

3. Verstraete K*, et al.* Structural insights into the extracellular assembly of the hematopoietic Flt3 signaling complex. *Blood* **118**, 60-68 (2011).

4. Verstraete K, Savvides SN. Extracellular assembly and activation principles of oncogenic class III receptor tyrosine kinases. *Nat Rev Cancer* **12**, 753-766 (2012).

5. De Smet F, Christopoulos A, Carmeliet P. Allosteric targeting of receptor tyrosine kinases. *Nat Biotechnol* **32**, 1113-1120 (2014).

6 Verstraete K*, et al.* Efficient production of bioactive recombinant human Flt3 ligand in E. coli. *Protein J* **28**, 57-65 (2009).
